# Development and Application of an MRT-qPCR Assay for Detecting Coinfection of Six Vertically Transmitted or Immunosuppressive Avian Viruses

**DOI:** 10.3389/fmicb.2020.01581

**Published:** 2020-07-17

**Authors:** Xiao Li, Keran Zhang, Yu Pei, Jia Xue, Sifan Ruan, Guozhong Zhang

**Affiliations:** Key Laboratory of Animal Epidemiology of the Ministry of Agriculture, College of Veterinary Medicine, China Agricultural University, Beijing, China

**Keywords:** MRT-qPCR, vertically-transmitted disease, immunosuppressive disease, coinfection, detection, application

## Abstract

Marek’s disease virus (MDV), reticuloendotheliosis virus (REV), avian reovirus (ARV), chicken infectious anemia virus (CIAV), infectious bursal disease virus (IBDV), and fowl adenovirus (FAdV) are important causes of disease in poultry. To investigate the infection status of the above six viruses in chickens in China, 1,187 samples from chicken flocks were collected and tested using a newly developed multiplex reverse-transcription quantitative real-time PCR (MRT-qPCR) assay in the study. A series of validation tests confirmed that the MRT-qPCR assay has high specificity, sensitivity, and repeatability. As for six detected pathogens, CIAV had the highest detection ratio, while ARV was not detected in any samples. In the spleen samples, the coinfection rate for MDV and CIAV was 1.6%, and that for REV and CIAV was 0.4%. In the bursa samples, the coinfection rate for FAdV and CIAV was 0.3%, and that for IBDV and CIAV was 1%. In the thymus samples, the coinfection rates for MDV and CIAV and for REV and CIAV were both 0.8%. Our study indicates that the coinfection of these viruses was existing in chickens in China. Through the detection of clinical samples, this study provides data on the coinfections of the above six pathogens and provides a basis for the further study of viral coinfection in chickens.

## Introduction

Immunosuppressive diseases not only cause disease but also weaken the immune system of the host, thus lowering the immune response and causing vaccinations to have a minimal effect (Adair, 2000; Sharma et al., 2000; Boodhoo et al., 2016; Shah et al., 2017; Gimeno and Schat, 2018). Some immunosuppressive viruses can also be transmitted vertically from breeding chickens to commercial generations, which makes the prevention and control of these diseases even more difficult (Witter and Salter, 1989). In recent years, some epidemiological surveys have shown that vertically transmitted or immunosuppressive diseases affecting chickens are widely distributed in most areas of China (Teng et al., 2011; Zhuang et al., 2015; Yang et al., 2017; Niu et al., 2018). These avian viruses can cause both single-virus infection and multiple-virus infection. Infections with multiple viruses complicate the pathogenesis, identification, prevention, and control of these pathogens (Li et al., 2017; Sun et al., 2017; Zhang et al., 2017; Cong et al., 2018). A rapid and efficient detection method is urgently needed to investigate the infection condition of these avian viruses.

A variety of alternative detection approaches have been described (Broeders et al., 2014; Cong et al., 2018; Garrido-Maestu et al., 2019). Real-time quantitative polymerase chain reaction (qPCR) provides an accurate, cost-effective, and high-throughput method for multiplex detection. Compared with the conventional PCR method, qPCR has higher specificity and sensitivity. It is a routinely used method for the detection and quantification of the gene in real time. Furthermore, qPCR can effectively detect multiple pathogens in one tube. Multiplex qPCR requires the use of probe-based assays, in which each probe is labeled with a unique fluorescent dye, resulting in different observed colors for each assay. Many RT-qPCR methods have been developed, providing useful references for researchers working on the diagnosis of avian viral infections (Luan et al., 2016; Wittwer, 2017; Laamiri et al., 2018).

Here, we established a probe-based multiplex reverse transcription-qPCR (MRT-qPCR) method to detect six vertically transmitted or immunosuppressive avian viruses: Marek’s disease virus (MDV), reticuloendotheliosis virus (REV), avian reovirus (ARV), chicken infectious anemia virus (CIAV), infectious bursal disease virus (IBDV), and fowl adenovirus (FAdV). The newly developed method was evaluated for its specificity, sensitivity, and repeatability, and then 1,187 samples from chicken flocks were collected and tested using the novel MRT-qPCR assay. The objective of the present study is to understand the infection and coinfection status of these six economically important avian pathogens.

## Materials and Methods

### Virus Strains and Clinical Samples

The strains of MDV, REV, ARV, CIAV, FAdV, and IBDV used in this study were isolated previously and are preserved in our laboratory. Clinical samples were collected from chickens from large-scale farms and free-range farmers all over China. These samples were initially brought to the Diagnostic & Research Center of Livestock and Poultry Epidemic Diseases, China Agricultural University, for the surveillance of other diseases. Samples were collected during 2017 and 2019 from varied ages and breed chickens, which consisted of broiler, layer, and native Chinese chickens. We selected three immune organs of chickens for sampling. In total, 1,187 samples were used, comprised of 491 spleen samples, 300 bursa samples, and 396 thymus samples.

### Primer and Taqman Probe Design

Sequences that have been published in NCBI of the six target viruses were downloaded and then aligned with the MegAlign software program in the DNAstar software package (version 5.01, DNAstar, Madison, WI, United States). Based on this information, probes and primers were designed in the conservative region and tested with the BLAST program^1^. BLAST was used to check the specificity of the probes and primers against other closely related genome sequences. All the primers were synthetized by TsingKe Biological Technology (Beijing, China). Different probes and primers for MDV, REV, and ARV were tested to determine whether they could be used together in a multiplex assay. A similar test was also performed for the other group, which consisted of CIAV, FAdV, and IBDV.

### Viral Nucleic Acid Extraction and Reverse Transcription

Total DNA was extracted from the clinical samples using the TIANamp Genomic DNA Kit (Tiangen, Beijing, China) in accordance with the manufacturer’s directions. DNA was eluted in a final volume of 70 μl. Total RNA was extracted from the clinical samples using the RNAprep Pure Organ Kit (Tiangen). RNA was eluted in a final volume of 30 μl. Reverse transcription (RT) was performed in a final volume of 10 μl per reaction at 37°C for 15 min, 85°C for 5 s, using PrimeScript^TM^ RT Master Mix (Perfect Real Time; Takara, Beijing, China) in accordance with the manufacturer’s directions. Nucleic acids were stored at -20°C until further use.

### Generation of Recombinant Plasmids for Use as Standards

To establish a quantitative assay and evaluate the specificity, sensitivity, and repeatability of the multiplex assay, six target genes were cloned to generate standard plasmids for use as positive controls. Electrophoresis of the PCR products was performed, and the final PCR products were purified using the Gel Extraction Kit (Omega, Norcross, GA, United States) in accordance with the manufacturer’s directions. The target genes derived from gel extraction were separately ligated with the pEASY-Blunt Cloning vector (TransGen, Beijing, China). The ligated products were transformed into *Escherichia coli* DH5α Competent Cells (Takara). The resulting recombinant clones were identified by sequencing. The recombinant plasmids were extracted using a Plasmid Miniprep Plus Purification Kit (Tiangen) in accordance with the manufacturer’s directions. The DNA concentration of each plasmid was spectrophotometrically determined with the Thermo Scientific NanoDrop 2000 spectrophotometer. The copy number of each extracted plasmid, equivalent to the number of viral genome copies, was calculated using the following formula (Huang et al., 2009):

$$\mathrm{Plasmid}\,\mathrm{copies}/\mu \,\text{l}=\frac{\left(6.02\times 10^{23}\right)*\left(\text{ng}/\mu \,\text{l}\times 10^{-9}\right)}{\mathrm{Plasmid}\,\mathrm{length}\,\left(\mathrm{bp}\right)\,660}$$

### RT-qPCR Assays

In both the single-plex reverse transcription-qPCR (SRT-qPCR) and the MRT-qPCR assays, the two-step reaction was performed with Premix Ex Taq^TM^ (Probe qPCR; Takara). The SRT-qPCR assay was carried out in a reaction system containing 10 μl of 2 × Premix Ex Taq^TM^, 3 pmol of each forward and reverse primers, 3 pmol of each probe, 2 μl of template, and 7.1 μl of nuclease-free water to reach a final reaction volume of 20 μl. For the MRT-qPCR assay, the reaction components consisted of 10 μl of 2 × Premix Ex Taq^TM^, 3 pmol of each forward and reverse primers, 3 pmol of each probe, 2 μl of template, and 5.3 μl of nuclease-free water to reach a final reaction volume of 20 μl. Each RT-qPCR assay was performed using a LightCycler^®^ 96 (Boehringer, Haftung, Germany) with the following procedure: 30 s of preincubation at 95°C, followed by 40 cycles of denaturation at 95°C for 5 s, and annealing and extension at 57°C for 30 s. The fluorescence emitted by different dyes was collected at the end of the annealing phase and analyzed using LightCycler^®^ 96.

### Sensitivity of the MRT-qPCR Assay

To evaluate the sensitivity of the MRT-qPCR assay, the MRT-qPCR and SRT-qPCR results were compared. The standard plasmid for each target gene was serially diluted 10-fold in Easy Dilution (TsingKe) to seven concentrations ranging from 10^9^ copies/μl to 10^3^ copies/μl. The diluted plasmids were then assessed using the SRT-qPCR and MRT-qPCR assays, and the resulting data were analyzed to obtain the single-plex and multiplex standard curves, respectively. The sensitivity of the MRT-qPCR assay was verified by comparing the single-plex and multiplex standard curves corresponding to the standard plasmid of each virus.

### Specificity of the MRT-qPCR Assay

Four steps were used to evaluate the specificity of the MRT-qPCR assay. First, BLAST was used to check whether the selected primers and probes matched other common avian virus. Second, the selected primers were synthesized and used in PCR, and the resulting products were analyzed via gel electrophoresis. Third, other avian viral genomes available in our lab were tested using the MRT-qPCR assay. Lastly, plasmids of different concentrations were mixed to form multiple combinations to detect whether there was competitive inhibition between different plasmids.

### Repeatability and Reproducibility of the MRT-qPCR Assay

To evaluate the repeatability and reproducibility of the MRT-qPCR assay, equal concentrations of standard plasmids for each of the three target viruses in each set were mixed together. Three different dilution levels (high-dilution, medium-dilution, and low-dilution) of the standard plasmid mixtures were prepared: 10^8^, 10^6^, or 10^4^ copies/μl, respectively. For intra-assay, each dilution of the positive plasmid was analyzed in triplicate. For inter-assay, each dilution was analyzed in three independent reactions. The coefficients of variation (CV) of the intra-assay and inter-assay were assessed to determine the repeatability and reproducibility of the MRT-qPCR.

### Detection of Clinical Samples

DNA and RNA were extracted from 1,187 samples, and the RNA was reverse-transcribed to obtain cDNA. Equal volumes of DNA and cDNA from the same tissue sample were mixed as a template. The MRT-qPCR assay described above was used to detect the nucleic acids of six specific viruses. The detection results of each organ were first analyzed separately. The single infection rate for each virus and coinfection rates for different combinations of viruses were determined. The data from the different organs were then compared with each other. The data from these experiments were also compared with the results of previously published epidemiological investigation reports.

### Statistical Analysis

In this study, the Spearman’s test is used to analyze the correlation between Ct values of the SRT-qPCR assay and the MRT-qPCR assay. All tests were two-sided, and statistically significant differences were assumed when *p* < 0.05. The CV is the absolute value of the ratio between the standard deviation (SD) and the mean (x):

$$C\,V=\frac{S\,D}{x}*100\%$$

## Results

### Qualities of the Positive Control Standard Plasmids

In this study, six positive control standard plasmids, each containing the target sequence of the given virus, were constructed and sequenced. When the BLAST reports generated from these sequences were analyzed, they showed that the inserted gene sequences of each plasmid were consistent with the target gene sequences. This indicates that each target gene was successfully transferred into the corresponding plasmid and that these positive control plasmids were suitable for further use.

### Sensitivity of the MRT-qPCR Assay

**FIGURE 1:**
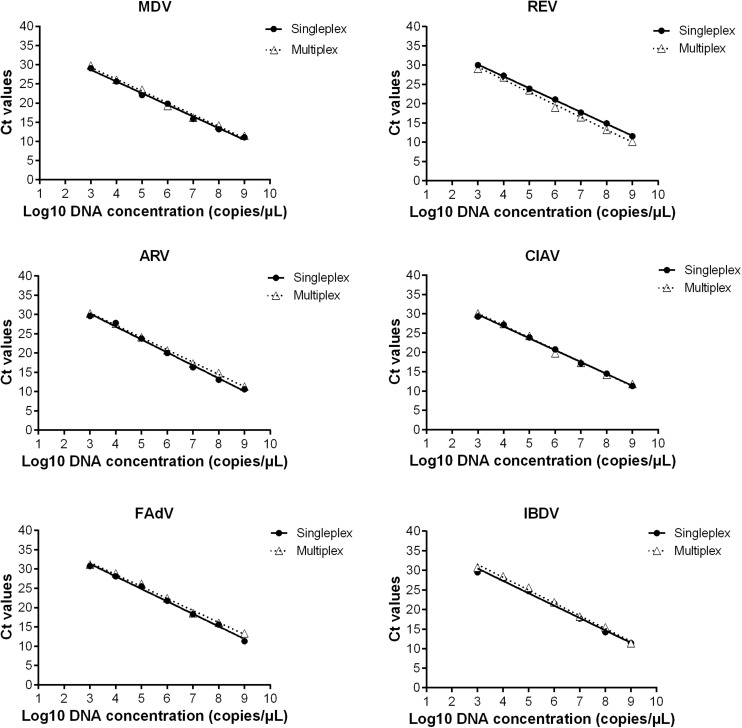
Standard curves from the SRT-qPCR and MRT-qPCR assays for MDV, REV, ARV, CIAV, FAdV, and IBDV. The horizontal axis displays the log_10_ number of plasmid copies. The vertical axis displays the mean Ct values from the SRT-qPCR and MRT-qPCR assays for each virus.

The sensitivities of the MRT-qPCR and SRT-qPCR assays were compared using standard plasmids that were serially diluted 10-fold from 10^3^ to 10^9^ copies/μl. The resulting standard curves for MDV, REV, ARV, CIAV, FAdV, and IBDV are shown in Figure 1. In each graph, the horizontal axis displays the log_10_ number of plasmid copies, and the vertical axis displays the mean Ct values produced by the SRT-qPCR and MRT-qPCR assays for each virus. The two standard curves (from the SRT-qPCR and MRT-qPCR assays) for each virus were relatively consistent with one another. The slope, Y-intercept, correlation coefficient (R^2^), and amplification efficiency (E) of the standard curves from the SRT-qPCR and MRT-qPCR assays are shown in Table 1. The slopes from the SRT-qPCR assay ranged from -3.04 to -3.36, and the slopes from the MRT-qPCR assay ranged from -3.07 to -3.27. The amplification efficiency was calculated with the equation E = (10^(–1/k)^) – 1, where k is the slope of the linear regression line (Kubista et al., 2006; Rutledge and Stewart, 2008). The *E*-values from the SRT-qPCR assay ranged from 98 to 113%, while the *E*-values from the MRT-qPCR assay ranged from 102 to 112%. All the *R*^2^-values from the SRT-qPCR and MRT-qPCR assays were greater than 0.98, which was considered acceptable (Broeders et al., 2014). A comprehensive regression analysis comparison confirmed that all six target viral genes were consistent between the data from the SRT-qPCR assay and that of the MRT-qPCR assay. There was no significant variation in the mean Ct values of the SRT-qPCR and the MRT-qPCR assays (*p* < 0.05), indicating that the two assays had good consistency. Hence, both assays may be considered equally sensitive.

**TABLE 1:** The sensitivities of the SRT-qPCR and MRT-qPCR assays, based on standard curves.

**Virus**	**The SRT-qPCR**				**The MRT-qPCR**			
	**Slope^a^**	**Y-intercept^b^**	***R*^2c^**	***E*^d^**	**Slope**	**Y-intercept**	***R*^2^**	***E***
MDV	–3.04	37.79	0.9946	113.27	–3.07	38.52	0.992	111.71
REV	–3.25	39.24	0.9949	103.09	–3.09	39.40	0.9994	110.68
ARV	–3.36	40.32	0.9935	98.44	–3.19	40.03	0.9986	105.82
CIAV	–3.15	39.62	0.9944	107.71	–3.08	39.05	0.9974	111.19
FAdV	–3.24	41.06	0.9956	103.54	–3.10	41.01	0.995	110.17
IBDV	–3.17	39.98	0.9926	106.76	–3.27	41.31	0.995	102.21

*^*a*^Slopes of standard curves. ^*b*^The intersection of the standard curve and the y-axis. ^*c*^Correlation coefficients (R^2^) of standard curves. ^*d*^Amplification efficiencies (E) of the SRT-qPCR and MRT-qPCR assays.*

### Specificity of the MRT-qPCR Assay

**FIGURE 2:**
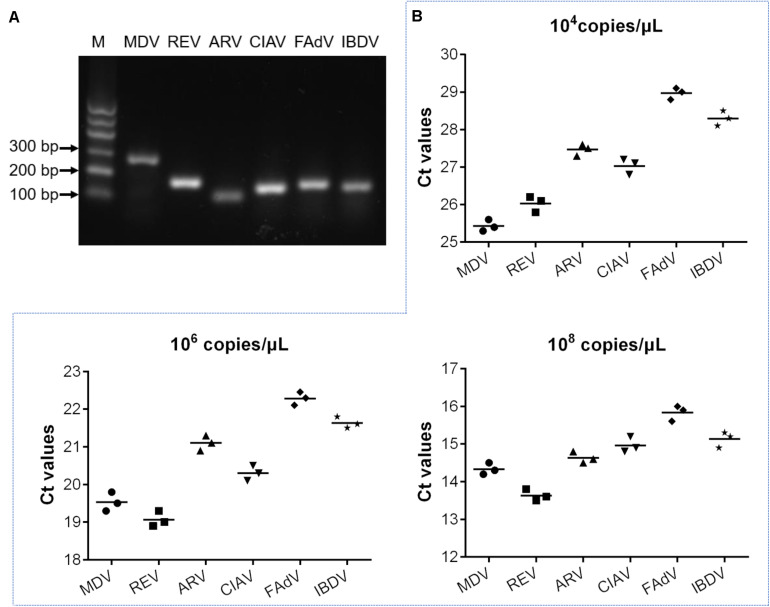
Detection of specificity and repeatability of the MRT-qPCR assay. **(A)** Gel electrophoresis of MRT-qPCR products produced with pairs of primers for MDV, REV, ARV, CIAV, FAdV, or IBDV. **(B)** Repeatability of the MRT-qPCR assay over three independent tests using three different mixtures of each standard plasmid. The high concentration mixture was composed of 10^8^ copies/μl of each standard plasmid, the medium concentration mixture was composed of 10^6^ copies/μl of each standard plasmid, and the low concentration mixture was composed of 10^4^ copies/μl of each standard plasmid.

All the primer and probe sequences listed in Table 2 were subjected to BLAST in NCBI. The results show that, for each sequence, the percentage of identical nucleotides with the target gene was up to 99%, and the percentage of identical nucleotides with other poultry pathogens was very low or non-existent. In addition, the specificity of these primers and probes was evaluated by the MRT-qPCR method. The six bands of MDV, REV, ARV, CIAV, FAdV, and IBDV visible in the agarose gel electrophoresis results are each consistent with the expected target band size (Table 2 and Figure 2A). The specific products were detected by sequence analysis. In terms of sequence alignment, the DNA sequencing results are consistent with the gene sequences of the target viruses, indicating that the selected primers and probes had specificity. We also performed MRT-qPCR tests on all the common avian pathogens available in our laboratory, such as avian avulavirus 1 (AAvV-1), avian influenza virus (AIV), infectious bronchitis virus (IBV), and avian infectious laryngotracheitis virus (ILTV). No fluorescence signals were collected, which indicated that this method only allowed the specific detection of the target gene. Finally, an evaluation test was conducted to detect whether the MRT-qPCR assay results were affected by competitive inhibition. Different combinations of low-dilution, medium-dilution, or high-dilution standard plasmids were used as test pools to evaluate the detection ability of the assay with multiple analytes, as in coinfection cases (Laamiri et al., 2018). The resulting Ct values from the different dilution mixtures of standard plasmid combinations varied with the plasmid concentration (Table 3). Together, these findings show that there was no competitive inhibition among the viruses in each group and that this method could detect changes in the template concentration.

**TABLE 2:** Primers and probes used in the MRT-qPCR assay.

**Virus**		**Probe/primer sequence (5′–3′)**	**Product size**
MDV	F	CCCACCACCTCCCATCT	258
	R	TGAGCGTAAACCGTCCC	
	Probe	FAM-TCTGCCCTCCCCAGCCTCCATCT- BQ1	
REV	F	CGTCATAAGAGGGCAGTCC	162
	R	ATTGATGGTCCCTGAAAGAG	
	Probe	Cy5-CACTTGCTGGTGGAACTGGGCTT- BQ2	
ARV	F	GGCCTMTCTAGCCACACCT	99
	R	TGGAGRTCGATTCGAGGTT	
	Probe	ROX-TTCTCGYATTACCGCCTTAGATCGT-BQ2	
CIAV	F	ATCAACCCAAGCCTCCCT	145
	R	CTCGTCTTGCCATCTTACAG	
	Probe	Cy5-TACCACTACTCCCAGCCGACCCC-BQ2	
FAdV	F	AAAACTGAGACTTTCCCACAA	163
	R	AGATACCCTCCGAAGAACTAC	
	Probe	HEX-TCTCCCATATCATTTCCATGCCTCC-BQ1	
IBDV	F	ACAGATTGTTCCGTTCATAC	144
	R	ATTAGCCCTGACCCTGTG	
	Probe	FAM-TTGTTGGCATCAGAAGGCTCCGT-BQ1	

**TABLE 3:** Specificity of the MRT-qPCR assay.

**Mixture MDV, REV, ARV Copies/μl**	**Average Ct**			**Mixture CIAV, FAdV, IBDV Copies/μl**	**Average Ct**		
	**MDV**	**REV**	**ARV**		**CIAV**	**FAdV**	**IBDV**
10^8^, 10^8^, 10^8^	14.32	13.22	14.38	10^8^, 10^8^, 10^8^	14.26	15.61	15.47
10^8^, 10^8^, 10^3^	14.19	13.33	32.21	10^8^, 10^8^, 10^3^	14.23	15.37	30.75
10^8^, 10^3^, 10^3^	14.13	28.95	31.94	10^8^, 10^3^, 10^3^	14.18	31.25	30.78
10^8^, 10^3^, 10^8^	14.27	29.24	14.32	10^8^, 10^3^, 10^8^	14.07	31.18	15.36
10^3^, 10^8^, 10^8^	30.16	13.17	14.26	10^3^, 10^8^, 10^8^	30.12	15.22	15.29
10^3^, 10^3^, 10^8^	29.97	29.14	14.19	10^3^, 10^3^, 10^8^	30.22	30.87	15.41
10^3^, 10^8^, 10^3^	29.62	13.35	31.98	10^3^, 10^8^, 10^3^	29.94	15.35	31.06
10^3^, 10^3^, 10^3^	30.06	29.23	32.17	10^3^, 10^3^, 10^3^	30.05	30.96	30.88

### Reproducibility and Repeatability of the MRT-qPCR Assay

To evaluate the repeatability and reproducibility of the MRT-qPCR assay, we selected three dilutions of plasmid mixtures for detection. The three selected plasmid concentrations were 10^4^ copies/μl, 10^6^ copies/μl, and 10^8^ copies/μl. Three independent tests were performed for each dilution level of the mixed plasmids. Each test was performed in triplicate by a different operator at a different time and in a different place. The Ct values of each pathogen obtained in the three independent tests are shown in Figure 2B. The coefficients of variation for intra-assay ranged from 1.07 to 3.24%. The inter-assay variability was in the range of 0.43–1.13% (Table 4). These results show that the MRT-qPCR method has good repeatability and reproducibility in detecting templates of different concentrations.

**TABLE 4:** Intra-assay and inter-assay coefficient of variation.

**Mixture (copies/μl)**	**Coefficient of variation (%)**											
	**MDV**		**REV**		**ARV**		**CIAV**		**FAdV**		**IBDV**	
	**Intra-assay**	**Inter-assay**	**Intra-assay**	**Inter-assay**	**Intra-assay**	**Inter-assay**	**Intra-assay**	**Inter-assay**	**Intra-assay**	**Inter-assay**	**Intra-assay**	**Inter-assay**
10^8^	3.22	0.87	2.84	0.91	2.69	0.85	1.85	1.13	3.24	0.88	1.94	1.12
10^6^	2.94	1.05	1.85	0.84	2.12	0.77	1.88	0.80	1.21	0.73	1.10	0.58
10^4^	1.42	0.49	1.34	0.65	1.09	0.45	1.83	0.63	1.07	0.43	1.41	0.58

### Detection of Clinical Samples

**FIGURE 3:**
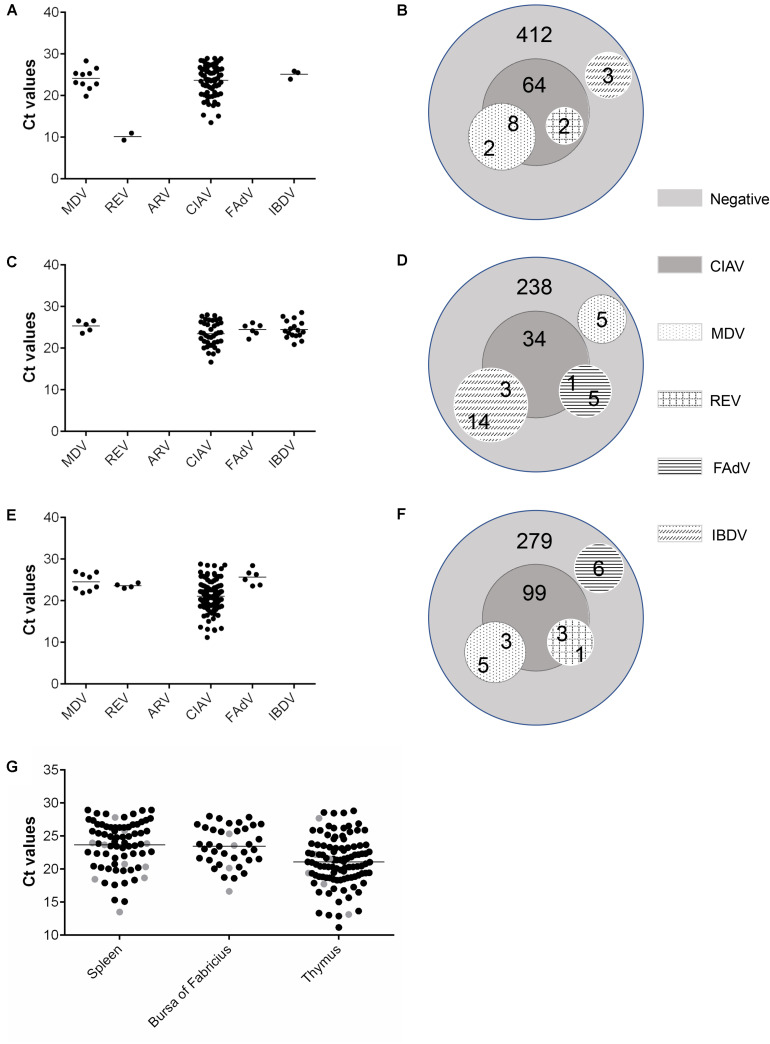
Virus detection results from the MRT-qPCR assay of the 1,187 clinical samples. For the spleen **(A,B)**, bursa of Fabricius **(C,D)**, and thymus **(E,F)** samples, the Ct value of positive samples for each virus are shown with scatter plots **(A,C,E)**. Each dot represents the mean Ct value of three duplicate in a positive sample. Coinfection samples are shown with Venn diagrams **(B,D,F)**. **(G)** Distribution of Ct values of CIAV in three tissues under simple infection and coinfection. Each dot represents the mean Ct value of three duplicates in a positive sample. The Ct values of CIAV single infection are shown with black dots. Coinfection of CIAV with different viruses is shown with gray dots.

A total of 1,187 samples, split into three groups based on sample type (spleen, bursa, and thymus samples), were assessed using the newly developed MRT-qPCR assay described above. From the overall analysis of these three groups of data, the highest detection rate was for CIAV, while ARV was not detected in any samples (Table 5). The Ct value of each positive sample is shown in Figures 3A,C,E. Coinfections of various combinations were composed of different viruses in conjunction with CIAV (Figures 3B,D,F).

**TABLE 5:** No. of positive samples and positive rate for six viruses in the spleen, bursa of Fabricius, and thymus.

**Tissues**	**No. of positive samples (positive rate)**					
	**MDV**	**REV**	**ARV**	**CIAV**	**FAdV**	**IBDV**
Spleen	10 (2.0%)	2 (0.4%)	–^a^	74 (15.1%)	–	3 (0.6%)
Bursa of Fabricius	5 (1.7%)	–	–	38 (12.7%)	6 (2.0%)	17 (5.7%)
Thymus	8 (2.0%)	4 (1.0%)	–	105 (26.5%)	6 (1.5%)	–

*^*a*^Not detected.*

Of the six tested viruses, only MDV, REV, CIAV, and IBDV were found in the 491 spleen samples; neither ARV nor FAdV was detected in any of these samples. Compared with the CIAV infection rate of 15.1%, the infection rates of MDV, REV, and IBDV (2, 0.4, and 0.6%, respectively) were relatively low. Only two coinfection types were observed in the spleen samples: MDV with CIAV (coinfection rate: 1.6%) and REV with CIAV (coinfection rate: 0.4%). 80% of MDV^+^ samples were also CIAV^+^. All REV^+^ samples were CIAV^+^ (Figure 3B).

In the 300 bursa of Fabricius samples, only MDV, FAdV, CIAV, and IBDV were detected; neither REV nor ARV was observed in any of these samples. As in the spleen samples, CIAV had the highest detection rate (12.7%). IBDV had the next highest detection rate (5.7%), followed by MDV and FAdV, with the relatively low detection rates of 1.7 and 2%, respectively. By comparing the detection rate of IBDV in three tissues, we can find that it has the highest positive rate in bursa of Fabricius. Of the seventeen IBDV^+^ samples, three were also CIAV^+^. Only one FAdV^+^ samples were also CIAV^+^ (Figure 3D). Coinfections of IBDV with CIAV (coinfection rate: 1%) and of FAdV with CIAV (coinfection rate: 0.3%) were present in the bursa samples.

Among the four viruses (MDV, FAdV, CIAV, and REV) detected in the 396 thymus samples, CIAV again had the highest detection rate (26.5%). The detection rates of MDV, FAdV, and REV were much lower and all very similar (2, 1.5, and 1%, respectively). ARV and IBDV were not detected in any of the thymus samples. Coinfections of MDV with CIAV and of REV with CIAV were observed in these samples. Both coinfections were detected at a rate of 0.8%. Three of eight MDV^+^ samples were MDV + CIAV coinfections in thymus samples, which was less than in spleen samples (eight of ten MDV^+^ spleen samples were also CIAV^+^) (Figure 3F). 75% of the REV^+^ samples were also CIAV^+^ in thymus samples while all of the REV^+^ samples were coinfected with CIAV in the spleen samples.

The positive rate of CIAV was the highest among the three tissues, and all the coinfection samples were CIAV with other viruses. Therefore, CIAV^+^ samples from the three tissue samples were analyzed separately. The samples of coinfection were marked with different colors to distinguish from CIAV single infection (Figure 3G). Ct values of CIAV from coinfection samples in three tissues were mostly concentrated near or below the mean value, indicating that the high viral load of CIAV *in vivo* could be conducive to the infection with other viruses.

## Discussion

Vertically transmitted or immunosuppressive diseases are economically important diseases of chickens and often cause great economic losses. Therefore, it is necessary to monitor the infection situation of such diseases in chickens. The MRT-qPCR assay established in this study, a two-reaction process, can detect six viruses known to cause vertically transmitted or immunosuppressive avian diseases (MDV, REV, ARV, CIAV, FAdV, and IBDV). The results from our specificity, sensitivity, and repeatability tests confirm that this novel detection method can specifically detect MDV, REV, ARV, CIAV, FAdV, and IBDV, respectively. The Ct values obtained by SRT-qPCR and MRT-qPCR assays performed with the same templates are very similar, indicating that the established MRT-qPCR assay is stable and applicable. Therefore, this method can be used as a diagnostic or research tool to monitor the clinical process of these virus infections. Once the genome has been extracted and reverse-transcribed, just two rounds of testing are needed to determine the positive vs. negative status of the six viruses in the sample. The establishment and application of this method will greatly lower the required reagent amount and performance time for testing, thus reducing the overall cost. Furthermore, multiplex assays have been widely applied for the detection of a large number of clinical samples (Laamiri et al., 2018). Thus, our newly established probe-based MRT-qPCR assay will be ideal for use in the routine analysis of clinical and experimental samples.

After validating the MRT-qPCR assay, we used it to investigate the positive rates of these six vertically transmitted or immunosuppressive viruses in chickens. 1187 clinical samples were obtained from the three main target organs: spleen, bursa of Fabricius, and thymus. Except for IBDV and ARV, the potential problem of vaccine interference is not applicable to these samples, and any pathogen detected in the organs can be considered as a natural infection. The positive rate of CIAV was between 12.7 and 26.5% after MRT-qPCR detection, while the positive rate of other viruses was below 5.7%. The positive rate of IBDV virus in the three tissues was significantly different. IBDV was detected only in the spleen and bursa of Fabricius samples. IBDV infects a variety of immune organs in chickens, but although its viral load varies widely in most organs, its positive rate remains stable in bursa (Kabell et al., 2005). This variability is the most likely reason for the observed difference in IBDV detection across the different tissues.

Previous studies showed that infection rates of CIAV, REV, MDV, and IBDV were 30.94, 4.16, 12.5, and 23.98% in sick and dead chickens in China (Wei et al., 2007). The results were consistent with our results in terms of the detection rate of each pathogen in the chicken flocks. We further confirmed that infection of CIAV was more frequent in the chickens. It has also been reported that chickens infected with FAdV are more likely to be infected with immunosuppressive diseases (Meng et al., 2018). Our results and existing epidemiological findings indicate that vertically transmitted or immunosuppressive diseases are prevalent in China (Eregae et al., 2014; Sun et al., 2017; Meng et al., 2018).

The coinfection of these diseases has great health problem to the poultry industry. The problem of coinfection is attracting more and more attention. In recent years, many researches about coinfection have been published (Miles et al., 2001; Eregae et al., 2014; Meng et al., 2018). However, no study has simultaneously investigated the coinfection of MDV, REV, ARV, CIAV, FAdV, and IBDV in chickens in China. In this study, the coinfection of the above six pathogens was detected. Through the MRT-qPCR identification of 1187 samples, results showed that coinfection is prevalent in chickens in China.

Immunosuppressive diseases are usually synergistic and complement each other’s immunosuppressive effects during coinfection. Our results showed that coinfection of viruses in all samples was CIAV with REV, MDV, IBDV, and FADV, respectively. Wei et al. (2007) found that the coinfection rate of CIAV and MDV was the highest in China. CIAV combined with MDV for coinfection increased the infectious capacity of MDV (Miles et al., 2001). CIAV also could assist FAdV in undermining the protection of maternal FAdV antibodies (Su et al., 2018). These results indicate that CIAV-infected chickens are more likely to be infected with immunosuppressive diseases, which is consistent with the results of our study. In the meantime, our results further show that when the viral load of CIAV *in vivo* is high, it is more favorable for coinfection with other viruses. Therefore, strengthening the prevention for CIAV and controlling the infection of CIAV could greatly reduce the occurrence of viral coinfections in chickens. Moreover, vaccine contamination problems have been reported in China and other countries, which cause exogenous viral infection and induce severe diseases (Woźniakowski et al., 2015; Li et al., 2017; Su et al., 2018). Our method can also be used to evaluate the safety of the vaccine, providing efficient protection for chickens.

## Conclusion

In conclusion, we successfully developed a reliable MRT-qPCR assay, which could detect six notable vertically transmitted or immunosuppressive diseases in chickens. By using this method, we investigate the positive rate of above six viruses in clinical samples. Results showed that coinfection is prevalent in chickens in China. This data could guide the future detection, prevention, and control of these diseases.

## Data Availability Statement

The raw data supporting the conclusions of this article will be made available by the authors, without undue reservation, to any qualified researcher.

## Author Contributions

GZ designed the study, supervised the study, and revised the final manuscript. XL, KZ, YP, and SR developed the method and performed the investigation. XL and JX wrote the manuscript. All authors read and approved the final manuscript.

## Conflict of Interest

The authors declare that the research was conducted in the absence of any commercial or financial relationships that could be construed as a potential conflict of interest.

## Footnotes

1 https://blast.ncbi.nlm.nih.gov/Blast.cgi
